# The Discussion on the Treatment of Placenta Prævia

**Published:** 1846-03

**Authors:** 


### PRACTICAL MEDICINE, &c.

*The Discussion on the treatment of Placenta Prævia.*— Within the present year, considerable space has, on various occasions, been occupied in the *Provincial Medical Journal*, and in the *Medical Gazette*, by a discussion on an important practical question in midwifery, viz: the treatment in unavoidable hæmorrhage from insertion of the placenta over the os uteri. Dr. Simpson, the able and learned professor of midwifery in the University of Edinburgh, proposed some years ago to supercede, in certain cases, the ordinary practice of puncturing the membranes, or turning; separating, instead, the placenta from the parieties of the uterus, and subsequently extracting it before the child; and he has since been zealously and ably advocating his views on this point. Between Professor Simpson and Dr. Radford, of Manchester, arose a question of priority, which was discussed at length in the *Provincial Journal;* and between Dr. Simpson and Dr. Lee has since arisen a controversy, firstly, with reference to the propriety of the new practice, and secondly, with reference to various statistical calculations. Hitherto we have remained silent, but attentive, spectators of the discussion, in the hopes of being able before long to lay some decided data before our readers. The question has, however, become so important, that we think it our duty to transfer to our columns a brief account of the principal points at issue, without, however, attempting for the present to give an opinion of our own. We shall, firstly, with reference to the question of priority, give a few extracts from the correspondence between Dr. Simpson and Dr. Radford, as published in the *Provincial Journal*, of February 5th; we shall then extract an admirable epitome of Professor Simpson's views published by him in a late number of the *Medical Gazette*, along with a counter-statement of Dr. Lee's. We shall not enter into any details respecting the statistical calculations, which have been the subject of much debate, as this would lead us into a part of the controversy which we wish to avoid. With the data which we now lay before our readers, they will be able perfectly to understand the main points of the question; and we trust that should our thus breaking the ground lead to any further discussion on the subject, it will be conducted in that calm and dignified manner in which scientific questions should always be treated.— *Lancet.*

*Dr. Simpson's remarks on the treatment of unavoidable Hæmorrhage by extraction of the Placenta before the child.*—All the more severe forms of uterine hæmorrhage that are liable to occur in the latter periods of pregnancy, and during delivery, are generally allowed, by obstetric pathologists, to depend upon the separation of a greater or less portion of the placenta from the interior of the uterus. When such a separation takes place, *two* surfaces are exposed, namely, *first*, a part of the inner surface of the uterus, and, *secondly*, the corresponding part of the outer, or maternal surface of the placenta. Both of these surfaces present a number of open vascular orifices left by the laceration of the utero-placental vessels which formerly connected them. From which set of open vascular orifices—the uterine or the placental—does the resulting hæmorrhage principally proceed.

Most accoucheurs seem to believe that the blood effused in those hæmorrhages which occur before or during labor, comes from the exposed *uterine* orifices. "It is (observes Dr. Lee,) from the great semi-lunar, valvular-like, venous openings in the lining membrane of the uterus, which you have seen in various preparations, and of [from] the arteries which are laid open by the separation of the placenta, that the blood *alone* flows in uterine hemorrhage."—(Lectures on Midwifery, p. 361.)

But arteries, particularly when they are so long and slender as the utero-placental arteries are, do not give rise to any marked degrees of hemorrhage when they are lacerated or *torn* through; and bleeding does not readily occur from the venous openings exposed on the interior of the uterus, because venous hemorrhage by *retrogression* (which the blood escaping backward into the uterine cavity would be) is here prevented by a variety of anatomical and subsidiary means, which I have elsewhere taken occasion to describe at some length.

In the passage that I have quoted above from Dr. Lee's published Lectures, Dr. Lee does not allow that the blood, in uterine hemorrhage, proceeds in any degree from the open venous orifices existing on the surface of the separated portion of placenta, the discharge proceeding, in his opinion, from the exposed *uterine* surface "alone." But I know of no reason, anatomical or otherwise, for alleging that the open *placental* orifices do not bleed; and, on the contrary, I believe with Dr. Hamilton and others, that the discharge issues principally or entirely from the vascular openings, which exist on that exposed placenta surface. These placental orifices are not, like the uterine, surrounded by contractile fibres capable of constricting them; they are in free communication with the general vascular system of the mother through the medium of the maternal vascular, or cavernous system of the placenta; and the blood in that cavernous system escapes readily from the exposed venous orifices on the surface of the placenta—that being, in fact, so far, its natural and *forward* course.

In cases in which the placenta is partially and repeatedly detached before labor begins (as happens frequently in placental presentations,) before each attendant attack of hemorrhage is arrested, the vascular system of the separated portion of placenta seems to require to become blocked up and impervious, with coagulated and unfiltrated blood. This obliteration of its vascular cells prevents the further circulation of maternal blood through the detached part of the organ, and hence prevents also the further escape of it from its exposed surface. Each new detachment gives rise to a renewed hemorrhage, which again ceases on the sealing up of the vascular system of the detached part. A few cases of placental presentation are on record in which there was *no* attendant hemorrhage when labor supervened, the tissue of the placenta having, throughout the whole organ, previously become so morbidly changed, obstructed, and impervious, as not to have any quantity of blood circulating in it and ready to escape, when at last its surface was separated from the interior of the cervix uteri under the occurrence of the uterine contractions.

In common cases of unavoidable hemorrhage, the amount of the attendant flooding seems to be as much regulated by the quantity of placental surface *still* remaining attached to the uterus, as by the quantity *already* separated from it—the degree of flooding depending as much or more, upon the extent of the means of supply of blood as upon the extent of its means of escape. And in proportion as we approach nearer and nearer a *total* separation of the placenta, the number of its *afferent* utero-placental vessels is diminished, till at last we find that when the one organ is once completely separated from the other, the flooding is instantly moderated, or entirely arrested; for the placenta ceases to yield any discharge of maternal blood as soon as its own supplies from the maternal system are thus cut off by the disseverment of all its organic and vascular attachments with the uterus.

Some years ago, I happened to see two cases of unavoidable hemorrhage, in which the placenta was spontaneously expelled for some hours, before the child itself was born. In both cases the attendant hemorrhage moderated, or entirely ceased, as soon as the whole placenta was completely detached. These instances, and others with which I was previously acquainted, forcibly suggested to my mind the idea that, under some complications in unavoidable hemorrhages, we might here (as in many other obstetric operations) adopt the principles of treatment at times successfully acted upon by nature herself, in her own unassisted management of such cases. I knew the fearful maternal mortality accompanying placental presentations, and that it was as great as, or even greater than, the fatality among patients attacked with yellow fever, or subjected to lithotomy. In order to ascertain if the *total* and complete detachment of the placenta afforded a greater chance of life to the mother, I collected and published in Dr. Cormack's Journal of Medical Science for March last, notices, which at that date I had brought together, of 141 cases of placental presentations in which the placenta was expelled or extracted before the child. The deductions which I ventured to draw from an analysis of these 141 cases were to the following effect:—

1. The *complete* separation and expulsion of the placenta before the child, in cases of unavoidable hemorrhage, is not so rare an occurrence as accoucheurs seem usually to believe; and it is not by any means so serious and dangerous as (according to the commonly received doctrines of uterine hemorrhage) might *à priori* be expected.

2. In nineteen out of twenty cases in which it has happened, the attendant hemorrhage was either at once altogether arrested, or became so much diminished as not to be afterwards alarming.

3. The presence or absence of flooding after the complete separation of the placenta does *not* seem in any degree to be regulated by the extent of the interval intervening between the detachment of the placenta and birth of the child.

4. In ten out of the 141 cases, or in one out of fourteen, the mother died after the complete expulsion or extraction of the placenta before the child; whilst, as we shall see immediately, about one in every three of the mothers die under turning and extraction of the child in unavoidable hemorrhage.

5. In seven or eight out of these ten natural deaths, the fatal result seemed to have no connection with the complete detachment of the placenta, or with consequences arising directly from it; and if we did admit the three remaining cases, (which are doubtful,) as leading by this occurrence to a fatal termination, they would still only constitute a mortality from this complication of three in 141,—or of about one in forty-seven cases.

These facts tend strongly to show that the artificial and complete detachment of the placenta would in all probability be in some cases and varieties, at least, of unavoidable hemorrhage, accompanied with much saving of maternal life. I know further, that in several instances recorded by Collins, Ramsbotham, Lowenhardt, &c., this treatment has been followed with success, when perchance it had been had recourse to midwives, and others, under supposed mismanagement, and in ignorance and defiance of all the established rules of practice in this special complication.

Exactly a year ago, I had an opportunity of putting, for the first time, to the test of experience, the practice which the foregoing remarks all lead to suggest, of *detaching, and, if necessary, extracting the placenta and not the child* in unavoidable hemorrhage. The lady (a patient of Mr. Hill, of Portobello,) was taken in labor between the seventh and eighth month of pregnancy, and, in consequence of the severity of the discharge, was blanched and prostrated when I first saw her.

The vagina was filled with coagula, and the os uteri was, in consequence of its small size and great height, reached and passed with difficulty, so as to ascertain fully the presentation of the placenta. Anterior to it I was able after a short time to reach and rupture the membranes. Notwithstanding this, however, along with the exhibition of ergot, &c., the discharge and sinking continued to go on. It seemed very difficult and dangerous to attempt to turn in consequence of the state of the os, and as the edge of the after birth was offering to protrude through it, I separated and gradually extracted the whole placental mass. From the time that this was accomplished all hemorrhage ceased. The cord was cut, and the placenta removed from the bed. The infant came down slowly, and was safely expelled about two hours afterwards. The mother made a perfect and speedy recovery.

Similar cases of the successful adoption of the same practice have, since the period at which my paper appeared in Dr. Cormack's Journal, been published by Mr. Wilkinson, Mr. Greenhow, Mr. Jones, and Dr. Maclean. In all these instances the mothers were saved, and rapidly recovered. Dr. Lever and Dr. Bird have informed me, within the last week, of two other recent successful instances of the same practice. In the course of a short time it seems not unreasonable to expect, that we may have a sufficient number of cases recorded, to enable us to judge with greater certainty and precision of the merits of this plan of treatment, and of the particular placental complications to which it may be especially applicable. *Medical Gazette.**

* We are obliged for want of space to postpone the remaining portion of this discussion until a future number.—[Ed.

